# Supplementary material for: Gut Microbiota and Serum Metabolome in Elite Cross-Country Skiers: A Controlled Study
Source: Metabolites. 2022 Apr 7;12(4):335. doi: 10.3390/metabo12040335 (PMC9028832; doi:10.3390/metabo12040335)
Supplement: Supplementary file 1 [file metabolites-12-00335-s001.zip › metabolites-1645411-supplementary.pdf]

SUPPLEMENTS

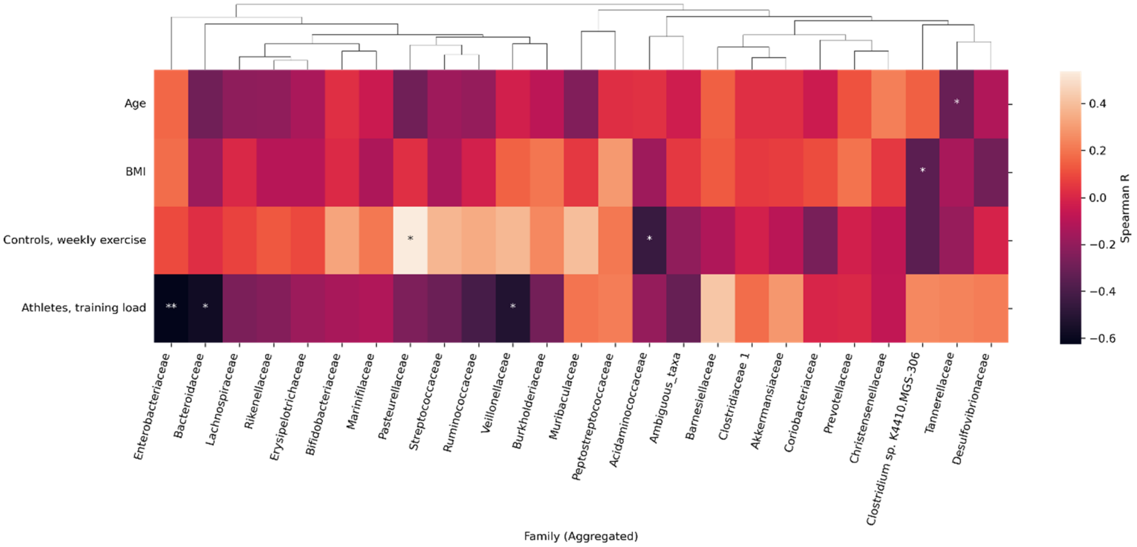

Figure S1. Clustered heatmap of Spearman correlation coefficients. between the bacterial families and background variables. Spearman *p*-value * < 0.05 ** < 0.01.

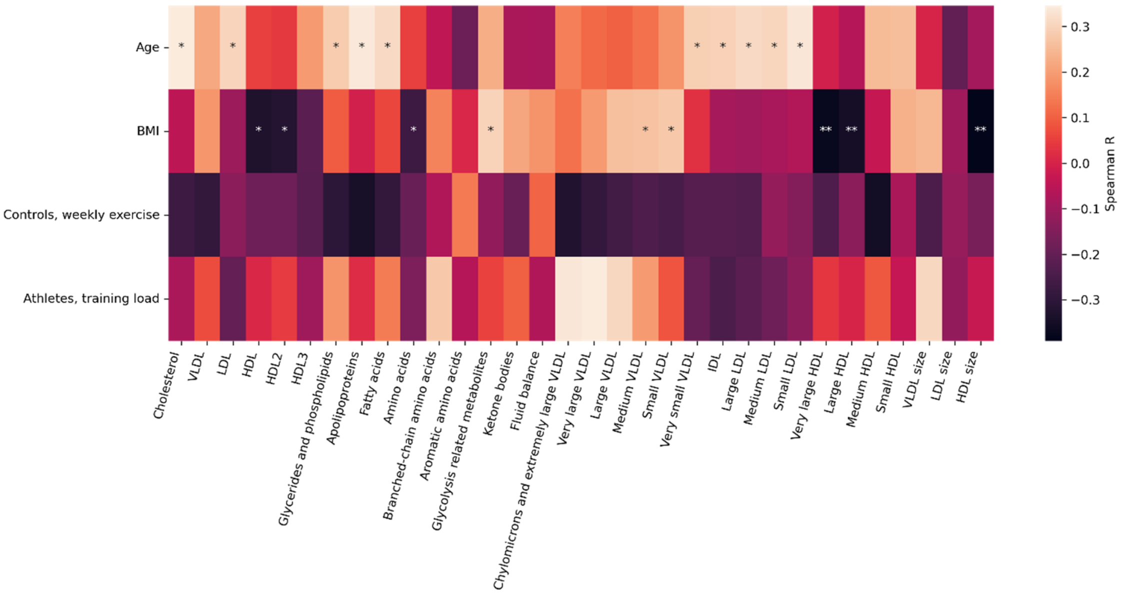

Figure S2. Heatmap of Spearman correlation coefficients between the metabolite groups and background variables. Spearman *p*-value * < 0.05 ** < 0.01.

Amino acids

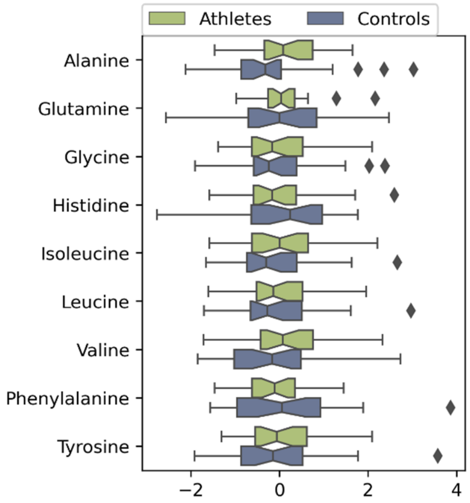

	Log2 fc	p	p adjusted
Alanine	0.071	0.08	0.109
Glutamine	0.029	0.801	0.946
Glycine	-0.014	0.853	0.706
Histidine	-0.026	0.391	0.364
Isoleucine	0.092	0.354	0.236
Leucine	0.066	0.511	0.317
Valine	0.092	0.195	0.113
Phenylalanine	-0.039	0.814	0.874
Tyrosine	0.036	0.48	0.474

Apolipoproteins

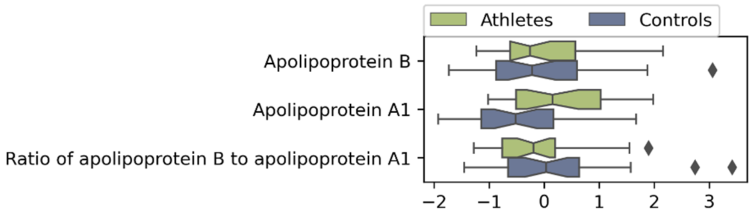

	Log2 fc	p	p adjusted
Apolipoprotein B	0.02	0.662	0.61
Apolipoprotein A1	0.135	0.009	0.023
Ratio of apolipoprotein B to apolipoprotein A1	-0.115	0.213	0.349

Cholesterol

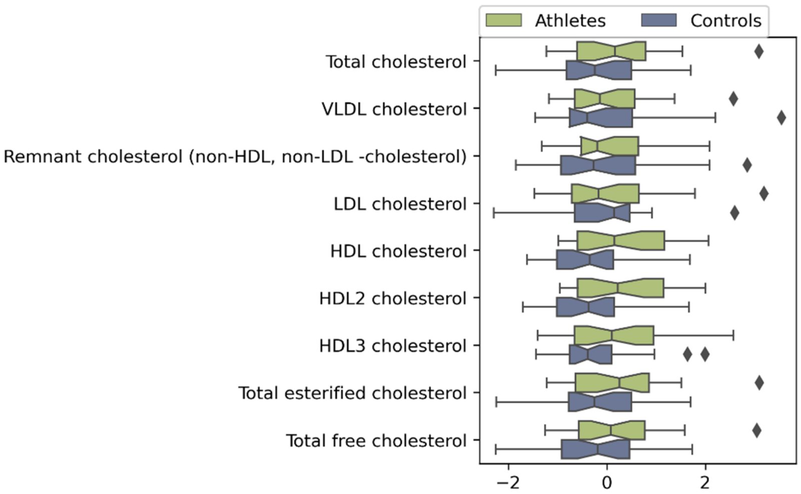

	Log2 fc	p	p adjusted
Total cholesterol	0.117	0.189	0.265
VLDL cholesterol	-0.013	0.801	0.596
Remnant cholesterol (non-HDL, non-LDL -cholesterol)	0.054	0.501	0.48
LDL cholesterol	0.049	0.814	0.944
HDL cholesterol	0.231	0.011	0.037
HDL2 cholesterol	0.319	0.01	0.034
HDL3 cholesterol	0.05	0.138	0.285
Total esterified cholesterol	0.124	0.173	0.24
Total free cholesterol	0.101	0.259	0.35

Fatty acids

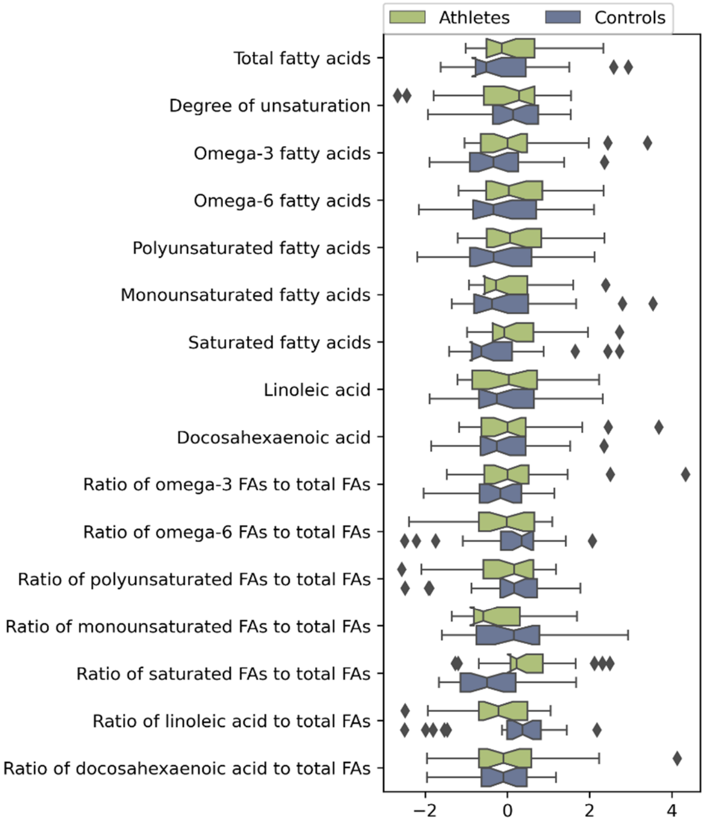

	Log2 fc	p	p adjusted
Total fatty acids	0.083	0.167	0.141
Degree of unsaturation	-0.02	0.602	0.434
Omega-3 fatty acids	0.181	0.232	0.234
Omega-6 fatty acids	0.065	0.297	0.314
Polyunsaturated fatty acids	0.077	0.252	0.254
Monounsaturated fatty acids	0.005	0.522	0.323
Saturated fatty acids	0.141	0.069	0.064
Linoleic acid	0.051	0.372	0.387
Docosahexaenoic acid	0.133	0.614	0.695
Ratio of omega-3 FAs to total FAs	0.116	0.469	0.557
Ratio of omega-6 FAs to total FAs	-0.035	0.297	0.14
Ratio of polyunsaturated FAs to total FAs	-0.02	0.637	0.393
Ratio of monounsaturated FAs to total FAs	-0.057	0.225	0.629
Ratio of saturated FAs to total FAs	0.059	0.001	0.002
Ratio of linoleic acid to total FAs	-0.055	0.059	0.026
Ratio of docosahexaenoic acid to total FAs	0.075	0.906	0.949

Fluid balance

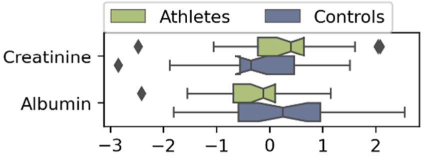

	Log2 fc	p	p adjusted
Creatinine	0.114	0.08	0.048
Albumin	-0.04	0.057	0.121

Glycerides and phospholipids

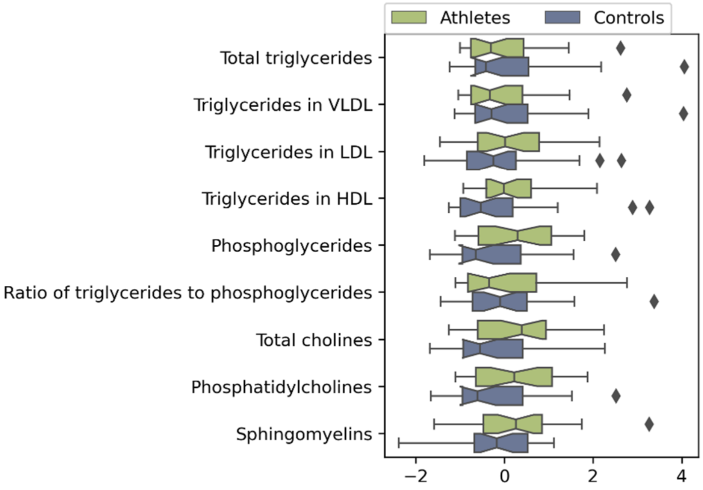

	Log2 fc	p	p adjusted
Total triglycerides	-0.067	0.92	0.745
Triglycerides in VLDL	-0.141	0.602	0.941
Triglycerides in LDL	0.101	0.252	0.249
Triglycerides in HDL	0.134	0.034	0.023
Phosphoglycerides	0.14	0.055	0.077
Ratio of triglycerides to phosphoglycerides	-0.069	0.501	0.887
Total cholines	0.128	0.055	0.089
Phosphatidylcholines	0.129	0.066	0.102
Sphingomyelins	0.131	0.092	0.164

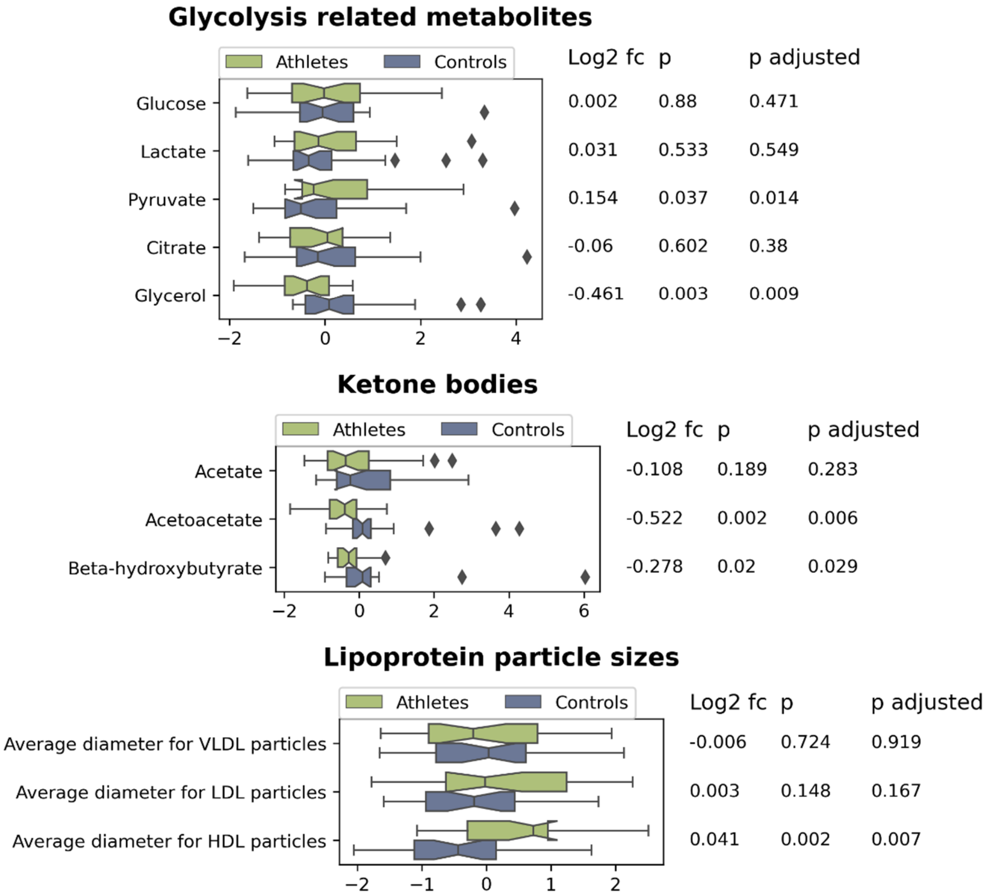

Figure S3. The metabolites and derived parameters. For boxplots, all values were mean centered. Whiskers indicate 1.5 times the interquartile range. Fold change between skiers and controls log2-transformed. *p*-values derived from Kruskal Wallis test. Adjusted *p*-values derived from Quade's ANCOVA adjusted for BMI and age.

Table S1. Results summary for linear regression model using *Butyricicoccus* and *Ruminococcus torques* group as dependent variables and associated metabolites as regressors.

Dep. Variable:	Butyricicoccus			
**F-statistic:**	82.88	**R-squared (uncentered):**	0.864	
**Prob (F-statistic):**	5.68E-17	**Adj. R-squared (uncentered):**	0.854	
	**Coefficient**	**Std error**	**t**	**P>|t|**
**HDL2 cholesterol**	0.767	0.509	1.507	0.14
**HDL size**	0.2151	0.066	3.26	0.002
**Acetoacetate**	-18.6707	7.912	-2.36	0.023
**Dep. Variable:**	[Ruminococcus] torques group			
**F-statistic:**	103.7	**R-squared (uncentered):**	0.838	
**Prob (F-statistic):**	1.49E-16	**Adj. R-squared (uncentered):**	0.83	
	**Coefficient**	**Std error**	**t**	**P>|t|**
**Acetate**	-13.756	11.906	-1.155	0.255
**Total cholesterol**	0.7533	0.116	6.499	0

Method: Ordinary least squares. R^2 is computed without centering (uncentered) since the model does not contain a constant. Standard Errors assume that the covariance matrix of the errors is correctly specified.
